# Possible Source of Intermediate Ions over Marine Environment

**DOI:** 10.1100/2012/425838

**Published:** 2012-06-04

**Authors:** Sunil D. Pawar, V. Gopalakrishnan

**Affiliations:** Thunderstorm Dynamics Program, Indian Institute of Tropical Meteorology, Pune 411 008, India

## Abstract

Measurements of small, intermediate and large ions made onboard ORV Sagarkanya over the Arabian Sea in May-June 2003 during Arabian Sea Monsoon Experiment (ARMEX) are reported here. The daily averaged values of small-, intermediate-, and large-ion concentrations measured for 36 days during this cruise have been used for analysis. The analysis shows a weak positive correlation of 0.14 between intermediate- and large-ion concentrations, which indicates that the sources of these two types of ions are different over ocean surface. The negative correlation is observed between small- and intermediate-ion concentration for entire period of cruise. In addition, it is seen that the intermediate-ion concentration shows a very good (*r* = 0.58) and significant positive correlation with sea surface pressure. Based on good negative correlation between small- and intermediate-ion concentrations and good positive correlation between intermediate-ion concentration and sea surface pressure, it has been proposed that attachment of small ions to the ultrafine particles transported from upper troposphere to marine boundary layer is the main source of intermediate ions over ocean surface. This study supports the idea that the main source of ultrafine particles over marine boundary layer (MBL) is entrainment of aerosol particles from the free troposphere.

## 1. Introduction

Atmospheric ions play an important role in determining the electrical state of the atmosphere. They determine not only the conductivity of air but also the influence of other atmospheric electricity parameters like air-earth current, atmospheric electric field, and space charge [1–3]. Cosmic radiation and radioactivity from ground surface are the major source of ionization over the ocean surface and land surface respectively. Air molecules attach themselves to primary ion pairs (single-charged positive ion and free electron) by ionization processes to form “cluster ions” known as small ions. Consequently, large and intermediate ions are formed by the attachment of small ions to uncharged aerosol particles. The main characteristic of air ion is electrical mobility, which is a function of its mass and size. In recent years, it has been recognized that, under some suitable environmental conditions, small ions grow to form ultrafine aerosol particles by the process known as ion-induced nucleation process [4–7]. Laakso et al. [8] have shown that the ion-induced nucleation process takes place when temperature, relative humidity (RH), concentrations of preexisting particles, and rate of ion production are favorable. Such conditions favorable for initiating ion-induced nucleation process exist in the upper troposphere and lower stratosphere [6]. Hõrrak et al. [4] and Vana et al. [9] have reported ultrafine particles bursts at three stations in Estonia and Finland. They attribute these bursts to the ion-nucleation process. Burst of nucleation mode particles has been reported over marine boundary layer also by Covert et al. [10] and Kamra et al. [11]. However, their contribution to ion nucleation has not been estimated.

In recent years, it has been recognized that atmospheric ions not only play important role in determining the electrical state of atmosphere but also can affect the aerosol and cloud properties and ultimately climate [5, 12–14]. Many studies have shown that electrical charge on aerosols can affect their dynamical properties. For example, coagulation rates and removal rate of aerosols by droplets can be greatly affected by electrical charge on aerosols, Clement et al., 1995 [15]. Moreover, many studies have shown that small ions can provide a source of atmospheric cloud condensation nuclei, which indicates a potential effect on clouds and ultimately climate [5, 13]. This has been supported by observations which show that variations in cosmic rays on the scales of days and years influence global cloudiness [16, 17]. Harrison and Carslaw [14] have emphasized that a mechanism linking cosmic ray ionization and cloud properties cannot be excluded and that there are established electrical effects on aerosol and cloud microphysics. They also emphasize the need of further work includes measurements of cloud, droplet, and aerosol charging and ion-aerosol conversion, together with modeling of the electrical aspects of nonthunderstorm cloud microphysics. 

 We report here our measurements of atmospheric ions classified as small, intermediate, and large ions based on mobility, made over Arabian Sea in May-June 2003 during Arabian Sea Monsoon Experiment (ARMEX). These are analyzed with respect to the observed sea surface pressure to examine the source of intermediate ions in the marine boundary layer.

## 2. Instrumentation

Hirsikko et al. [18] list various techniques used by many researchers for measurements of atmospheric ions. For example, Mitisen et al. [19] used integral air ion counters for their measurements at Tartu, Estonia. Kulmala et al. [20], Iida et al. [21], and Vanhanen et al. [22] used Condensation Particle Counter-Based Differential Mobility Analyser for measuring the air ions of diameter down to 1 nm. We used an ion counter similar to one described by Dhanorkar and Kamra [23], is used to measure the concentration of positive atmospheric ions in three different size categories based on their mobility. The ion counter consists of three Gerdien's condensers. A common fan is used to suck the air through these three condensers. The three condensers are designed to measure the concentrations of small, intermediate, and large ions in the mobility ranges of >0.77 × 10^−4 ^m^2^ V^−1^ s^−1^, 1.21 × 10^−6^–0.77 × 10^−4 ^m^2^ V^−1^ s^−1^, and 0.97 × 10^−8^–1.21 × 10^−6 ^m^2^ V^−1^ s^−1^, respectively. The ion counter is installed on the balloon-launching platform of ORV Sagarkanya at ~9 m above sea level with its intake perpendicular to the direction of ship's motion. Care has been taken to see that ship's chimney exhaust does not contaminate the measurements by positioning the inlet of apparatus in upwind of ship's exhaust. However, the data obtained during the periods when the measurements are visibly observed to be contaminated are not included in the analysis. Some of the observations made using this ion counter have already been reported in Pawar et al. [24].

## 3. Cruise Track and Meteorological Conditions

**Figure 1:**
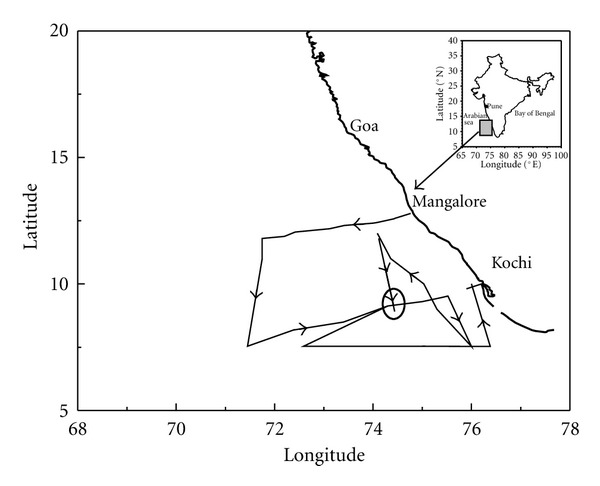
Cruise track of ORV Sagarkanya during the period from May 16, 2003 to June 19, 2003.

**Figure 2:**
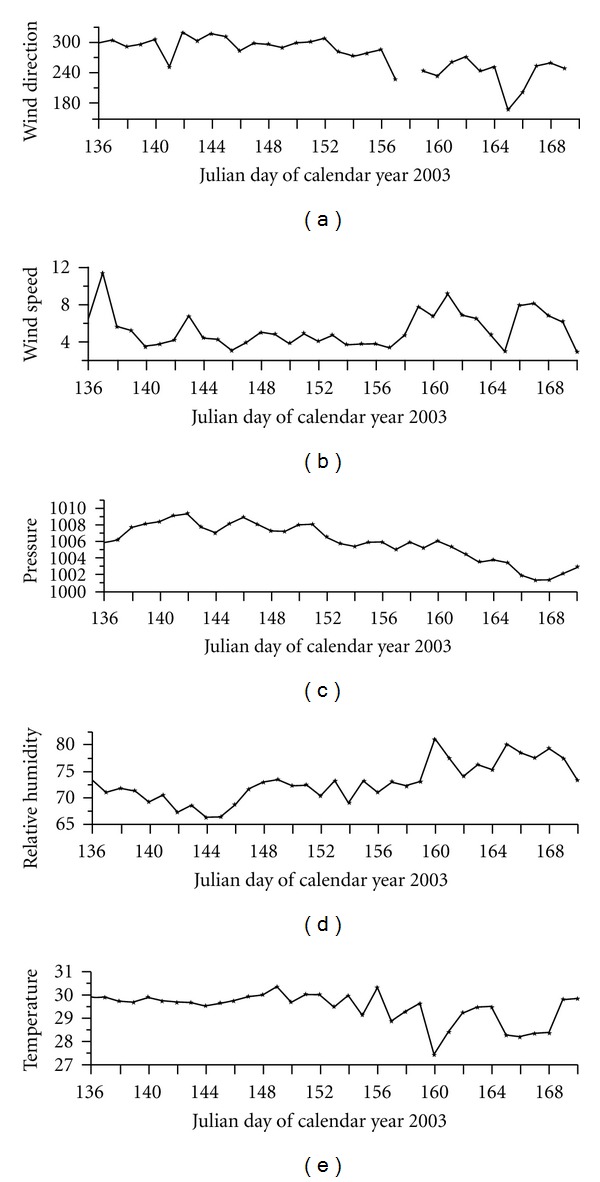
Daily averaged values of air temperature, relative humidity, atmospheric pressure, wind speed, and direction during the cruise period.

Figure 1 shows the cruise track of ship ORV Sagarkanya from May 16 to June 19, 2003. The cruise started from Mangalore, India (12.9°N, 74.8°E), on May 16, 2003 and sailed initially westwards and then southwards. The ship was kept stationary at around 9.2°N and 74.5°E from May 23 to June 7, 2003 to conduct time series observation of conductivity-temperature-depth (CTD). On June 7, ship started moving towards east and reached Kochi on June 19.  Figure 2 shows the daily averaged values of different meteorological parameters recorded onboard the ship. The atmospheric temperature was generally around 30°C except for some rainy days when it decreased to around 27-28°C. The relative humidity varied from 65% to 80% during cruise period. Winds, which were calm and westerly initially, became gradually strong and changed their direction to southwesterly with the onset of south-west June 7, 2003. Wind speed was between 4 to 9 m sec^−1^ during the cruise period. Sea surface pressure varied from 1002 mb to 1008 mb during this period. We report here only the observations about 50 to 400 km away from the Indian coast.

## 4. Observations

### 4.1. Relationship with Small and Large Ions

**Figure 3:**
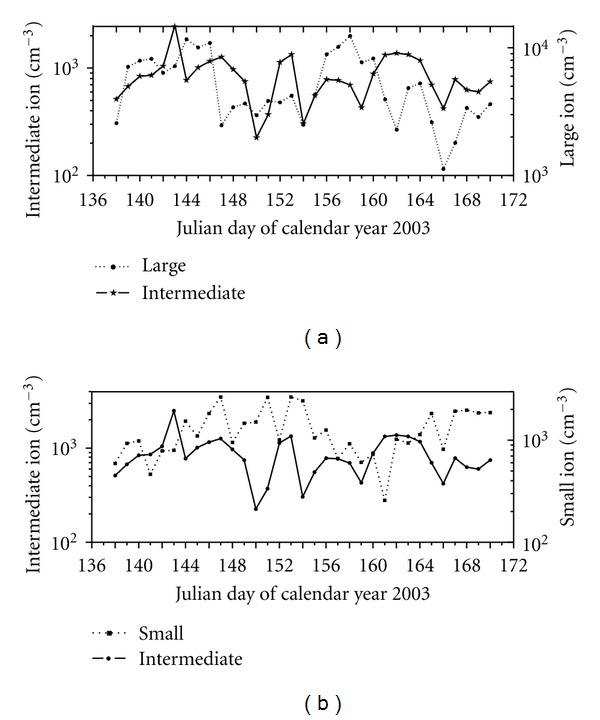
Daily averaged concentrations of small, intermediate, and large ions for the entire cruise period.

Figure 3 shows the daily average values of small, intermediate and large ions measured during whole cruise period. As shown in this figure, small-ion concentration lies in the range 300 to 3000 cm^−3^ with an average of 974 cm^−3^. On the other hand, large-ion concentrations show large variations over the cruise period and lie between 1000 and 10000 cm^−3^ with an average of 5528 cm^−3^. Intermediate-ion concentrations also show large day-to-day variation, and the concentrations vary from 200 to 3000 cm^−3^ with an average concentration of 877 cm^−3^. As shown in Figure 2, large-ion concentration shows overall decreasing trend with the progress of cruise and small-ion concentration shows increasing trend. However, intermediate-ion concentration does not show any trend during the cruise period. Further, the intermediate ions generally follow variations in large ions and vary inversely with small-ion concentration. It should be worth noting here that the inverse correlation between intermediate- and small-ion concentrations is enhanced for periods when large-ion concentration is less.

**Figure 4:**
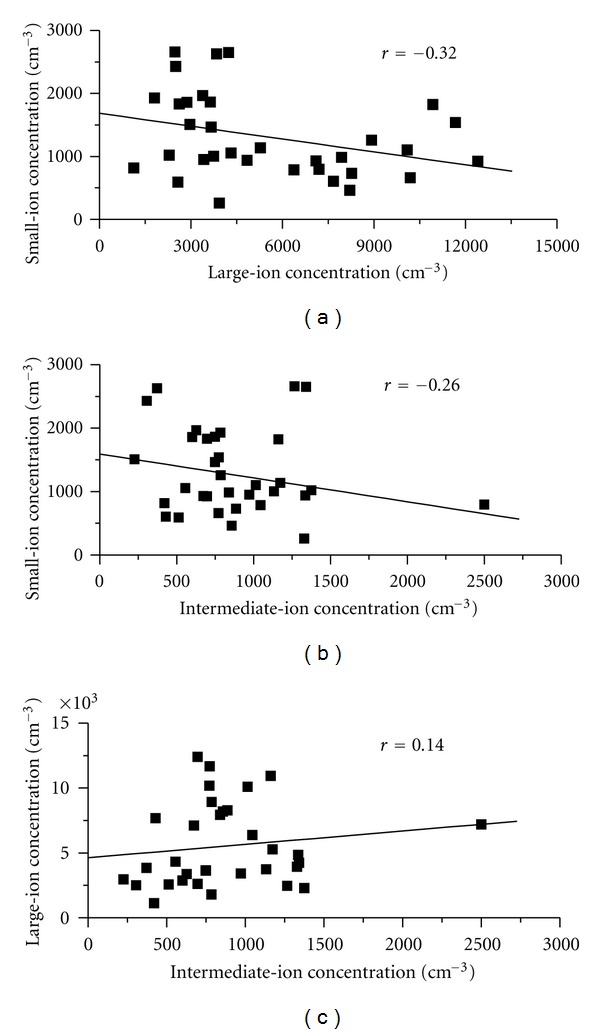
Scattered diagram of daily averaged (a) concentrations of large and small ions; (b) concentrations of intermediate and small ions; (c) concentrations of intermediate and large ions. Line of best fit is also shown.

**Figure 5:**
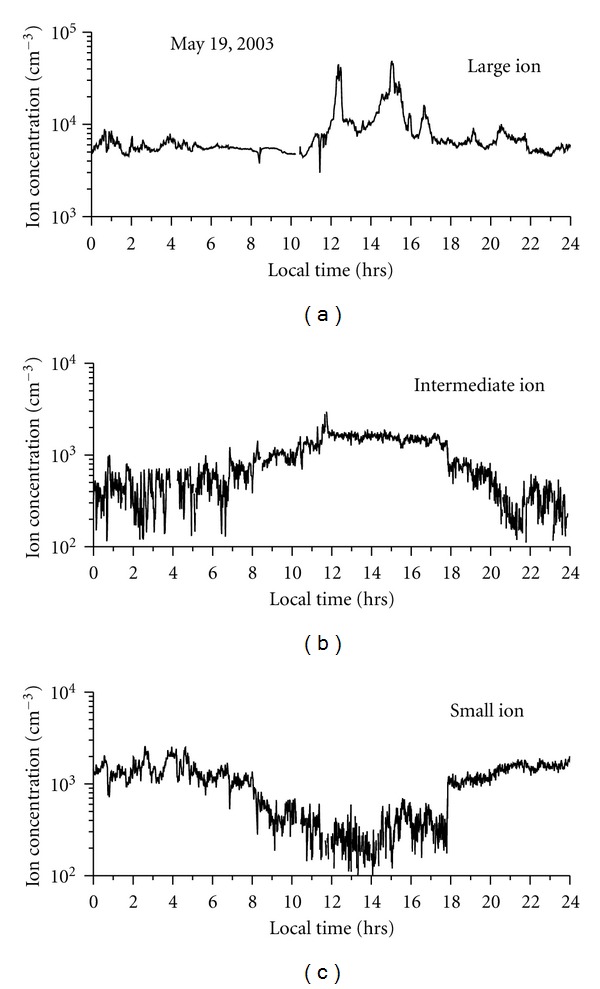
Diurnal variation of small, intermediate, and large ions on a fair weather day (May 19, 2003) observed during cruise period.

Figure 4 shows the scattered plot of intermediate-ion concentration versus small-ion (Figure 4(a)) and large-ion concentrations (Figure 4(b)). The correlation coefficient between intermediate ions and large ions is only 0.14, which indicates these two parameters are poorly correlated or sources and sinks of both types of ions can be different from each other over ocean surface. The inverse correlation between intermediate and small ions is also less (*r* = −0.26). The negative correlation between small ions and large ions is also not significant (Figure 5) which suggests that the attachment of small ions to uncharged aerosol particles may not be the main source of large ions over ocean surface. Further, Pawar et al. [24] have shown from National Centre for Environmental Prediction (NCEP) derived wind analysis that charged salt particles generated by wave breaking are the main source of this high concentration of large ions over this region.

### 4.2. Diurnal Variation

**Figure 6:**
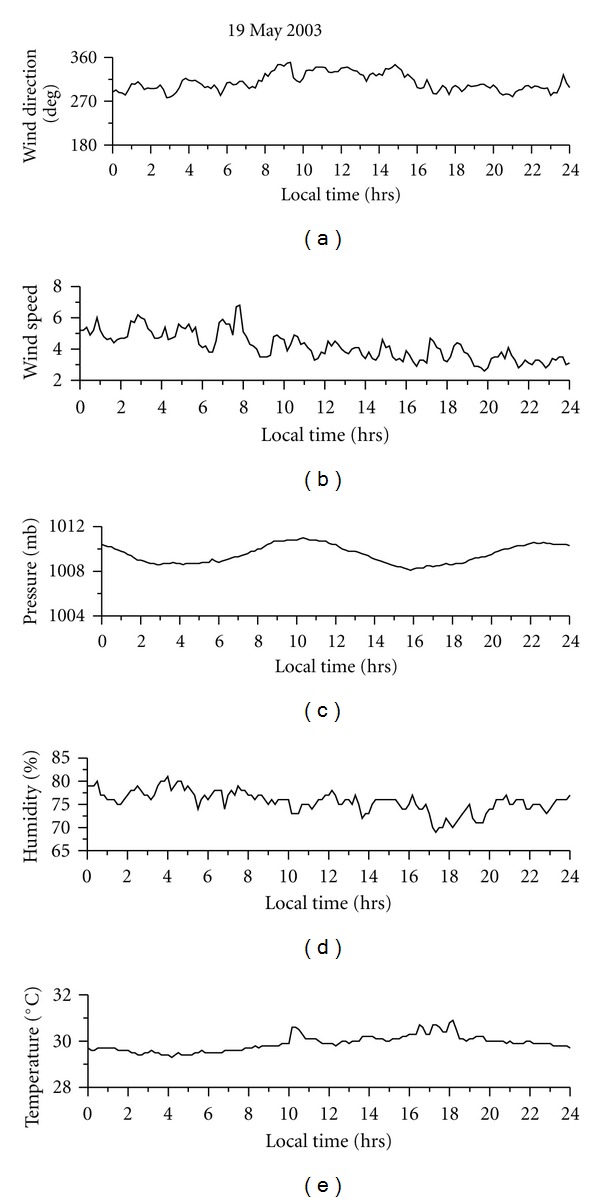
Diurnal variation of meteorological parameters on May 19, 2003.

**Figure 7:**
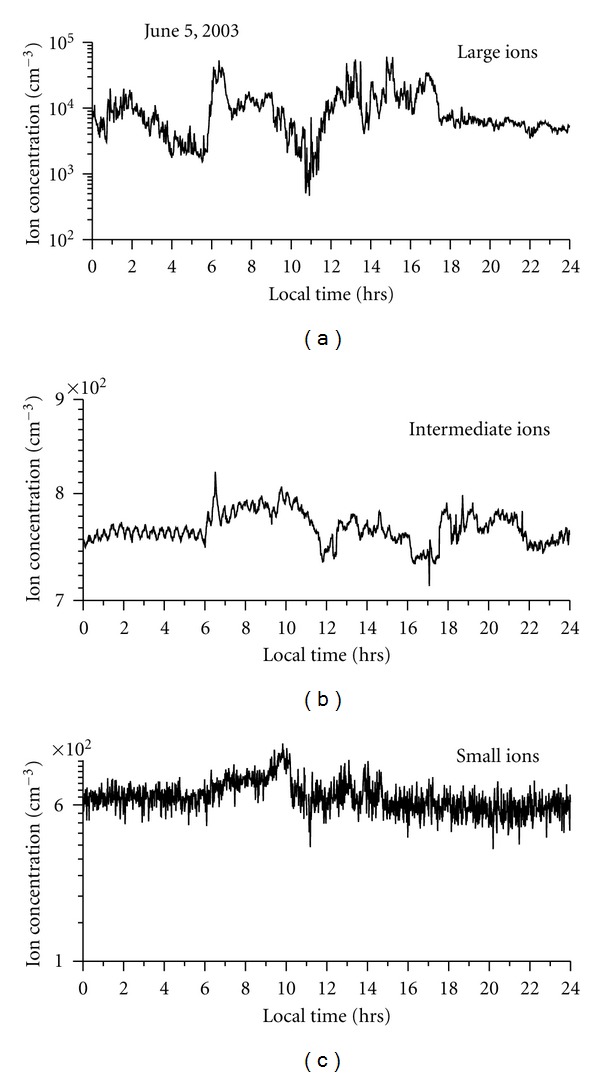
Diurnal variation of small, intermediate, and large ions on a cloudy day (June 5, 2003) observed during cruise period.

**Figure 8:**
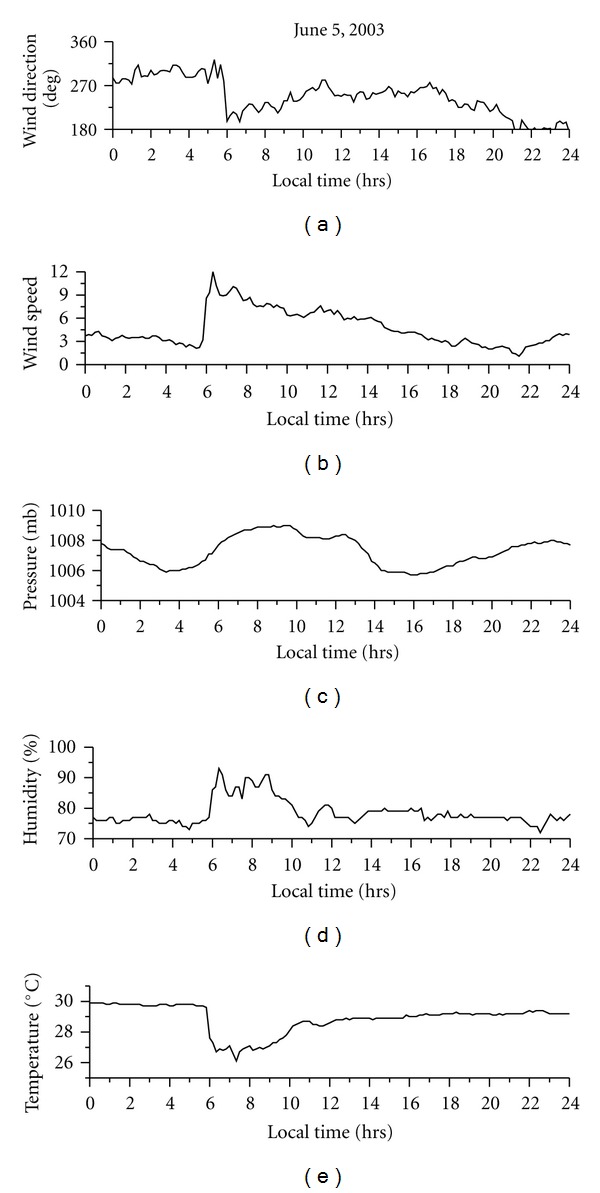
Diurnal variation of meteorological parameters on June 5, 2003.

Dhanorkar and Kamra [23] have shown that all three types of ions, that is, small, intermediate, and large, show higher concentrations during night compared to day. They attributed this increased ion concentrations to the increased ionization rate during night time due to increased radioactive gasses. The observations of Hõrrak et al. [25] at Tahkuse observatory in Estonia show that the concentration of small ions shows maxima in the early morning hours and minima in the evening hours. They also observed that the size distribution of intermediate and light large ions in the range of 1.6–22 nm is strongly affected by nucleation bursts of nanometer particles. On the burst days, the maximum concentration of intermediate ions (1.6–7.4 nm) is about the noontime and that of light large ions (7.4–22 nm) about 2 hours later. Hõrrak et al. [4] observed that the concentration of intermediate ions is strongly correlated with temperature during the diurnal cycle. There are no reports of diurnal variation of atmospheric ions over ocean. Figure 5 shows the diurnal variation of small, intermediate, and large in concentrations on a typical fair weather day, May 19, 2003. As shown in this figure, there is an increase in intermediate ions in the afternoon hours. The small-ion concentration shows decreases during same time. Large-ion concentrations also reach their maxima during the afternoon hours. Almost similar pattern of diurnal variation is seen on other fair-weather days also. The diurnal variation of associated meteorological parameters is shown in Figure 6. This day was a fair weather day with clear sky throughout the day. The winds were moderate and from west to west-northwest direction. The diurnal variation of small-, intermediate-, and large-ion concentrations on nonfair weather day is shown in Figure 7. On that day, sky was cloudy and there was moderate rain in the morning hours.  Figure 8 shows the associated meteorological parameters observed on that day. After the rainy spell, the atmospheric temperature was around 30°C and the relative humidity remained about 75%. There were winds exceeding 5 m sec^−1^ during the day and afterwards the winds were calm. As shown in Figure 7, intermediate-ion concentration does not show any diurnal variation as observed on fair weather day (Figure 6). There was a sharp increase in concentration of intermediate and large ions during rain. There was not much variation in small-ion concentration.

### 4.3. Relationship with Air Pressure

**Figure 9:**
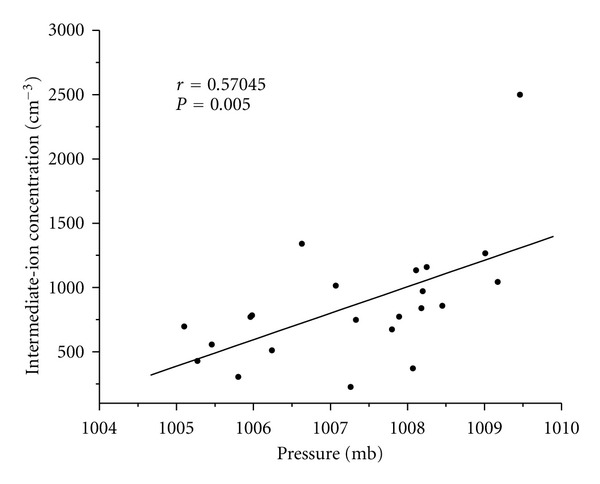
Scattered diagram of daily averaged intermediate-ion concentration and daily averaged sea surface pressure with line of best fit.

 The main source of ultrafine particles over marine boundary layer is transported from free troposphere [10, 26]. The observations of Pawar et al. [27] show such a transport of ultrafine particles can be controlled by sea level pressure. Our observations also show a significant positive correlation between daily averaged values of intermediate-ion concentrations and sea surface pressure (Figure 9). Many studies [6, 28] have shown that, in the free troposphere because of favorable conditions like high ionization rate, low background aerosol concentration, and low temperature, large number of ultrafine particles form via ion-induced nucleation process. The good correlation observed between intermediate ions and sea surface pressure suggests that the transport from troposphere is the main source of observed intermediate ions. It should be noted here that, in their life time of about few minutes, possibility of intermediate ions formed in the upper or middle troposphere reaching sea surface is much less. However, the possibility of transportation of intermediate ions formed in the lower troposphere or just above marine boundary layer to marine boundary layer cannot be ruled out.

## 5. Discussion

 The attachment of small ions to ultrafine particles is considered as one of the dominant processes for formation of intermediate ions in the atmosphere [23]. Our observations, which show inverse relationship between small ions and intermediate ions, support this idea (Figure 4). However, correlation between these two parameters is low and statistically insignificant mainly because of presence of aerosols bigger than ultrafine particles, which can also act as a sink for small ions. The concentration of large ions can give some indication about the concentration of aerosols bigger than ultrafine particles. To illustrate the idea that the main source of intermediate ions is the attachment of small ions to ultrafine particles, we have chosen two periods, from May 26 to June 3 (Julian day 147 to 155) and from June 10 to June 18 (Julian day 162–170), when large-ion concentrations are less (Figure 3). As the large-ion concentration are low, we can assume that aerosols bigger than ultrafine particles will also be less during these periods. During these two periods, a good, statistically significant inverse correlation up to 96% confidence exists between small-ion and intermediate-ion concentration. From this good and statistically significant inverse correlation observed between intermediate- and small-ion concentrations, it is proposed that attachment of small ions to ultrafine particles can be the main source of intermediate ions over ocean surface. The typical diurnal variations observed on fair weather and cloudy days support this idea (Figures 5 and 7). Small and intermediate ions show significant inverse correlation on fair weather day, whereas, on cloudy days, the correlation is positive. The inverse correlation between small and intermediate ions on fair weather day suggests that small ions have depleted by newly formed ultrafine particles. It is interesting to note that, on cloudy day, the correlation between these ions is positive, even though correlation coefficient is small, this correlation is significant up to 99.9%. Such a positive correlation suggests that the source of these two types of ions can be same during rainy day. Our observations of typical diurnal variation of intermediate and small ions on fair weather days as shown in Figure 4(c) and significant inverse correlation observed between them also suggest that source of intermediate ions is the attachments of small ions to the newly formed ultrafine particles by photochemical reactions on sunny days.

 The observations by Covert et al. [10] and Kamra et al. [11] and model calculations by Raes [26] and Yu et al. [29] show that the entrainment from free troposphere is a main source of the particles in nucleation mode in the marine boundary layer. Pawar et al. [27] have shown that the sea level pressure controls such transport of aerosol particles during subsidence motion from free troposphere to the marine boundary layer. The good positive correlation observed between surface pressure and intermediate ions (Figure 9) suggests that the one of the source of intermediate ions is attachment of small ions to the ultrafine particles transported from free troposphere.

The negligible correlation between large and intermediate ions suggests that source of these two types of ions can be completely different from each other. From the good positive correlation observed between the large-ion concentrations and area averaged NCEP-derived winds over Arabian sea, Pawar et al. [24] have suggested that the charged salt particles generated by wave breaking may be the main source of large ions over marine boundary layer.

## 6. Conclusions

Our observations strongly demonstrate that the attachment of small ions to the ultrafine particles is the main source of intermediate ions over marine boundary layer. The inverse correlation observed between small ions and intermediate ions in the daily averaged as well as in one-minute averaged data supports this idea. Our observations also demonstrate that the ultrafine particles are being transported from upper troposphere to marine boundary layer. The diurnal variation of intermediate ion on fair weather day which shows increased concentration in the noon time suggests that new particle formation by photochemical reaction may be one of the source of ultrafine particles over marine boundary layer. The poor correlation between intermediate and large ions suggests that the sources of these two types of ions can be completely different over marine boundary layer. Poor correlation between large ions and small ions indicates that attachment of small ions to aerosol particles may not be the main source of formation of large ions over marine boundary layer.
